# On Injuries of the Head Affecting the Brain

**Published:** 1843-01

**Authors:** 


### ART. XIV.

*On Injuries of the Head affecting the Brain.* By G. J. GUTHRIE, F.R.S. &c. &c.—*London*, 1842. 4to, pp. 155.

WE should but trifle with our readers, and employ our space most unprofitably were we to insist on the great practical importance of those affections which constitute the subject of Mr. Guthrie's treatise. We shall, on this point, content ourselves with quoting and adopting our author's opening sentence: "Injuries of the head affecting the brain are difficult of distinction, doubtful in their character, treacherous in their course, and for the most part fatal in their result." A commentary on such a theme written by a surgeon of experience and reputation cannot fail to attract the attention of the profession, and demands on our part a candid and careful examination, a duty which we shall best discharge by giving an analysis of Mr. Guthrie's treatise, accompanied by such remarks as, in our opinion, may seem called for. The reader can then judge for himself whether Mr. Guthrie has removed any of the difficulties, or cleared up any of the doubts, that so often occur to even the most consummate practitioner in the treatment of injuries of the head.

In the first few pages of the work Mr. Guthrie discusses the question of localization of disease or injuries of the brain; whether, for example, we can determine from the symptoms what part of the brain may suffer from compression, or be occupied by an abcess; and the same question is again considered at greater length when treating of compression of the brain. The physiological and pathological researches of Flourens, Mayo, Magendie, Bouillaud, Marshall Hall, &c. are given in brief yet sufficient detail so far as they bear on this subject, respecting which Mr. Guthrie coincides in opinion with every practitioner of sufficient experience, acknowledging that, "such are the deficiencies in our knowledge of the complicated functions of the brain, that although we think we can occasionally point out where the derangement of structure will be found, which has given rise to a particular symptom during life, the very next case may possibly show an apparently sound structure with the same derangement of function." In fact, if we attempt to base our diagnosis on the symptom of paralysis for example, how much more do we know than was ascertained by Hippocrates, who announced that the cause of mischief occupied the side of the brain opposite to the paralysed limb. The progress of science has merely taught us that the rule though general is not universal, as in some few cases the paralysis and the lesion of the brain exist on the same side: Mr. Guthrie states (p. 45) that Burdach has collected fifteen cases in which this occurred. We think, however, that M. Rochoux has shown that several of these cases are far from being conclusive, but it must be admitted that seven or eight of them establish the point beyond doubt, and Mr. Guthrie himself records a case (p. 26) in which, to use his own words, there was "rupture of the roof of the ventricle of the *right* or the *same* side of the body as that which was affected" with paralysis; though he does not allude to this remarkable case in connexion with this particular point. Foville and Pinel Grandchamp, as our readers must be aware, consider that paralysis of the upper extremity indicates a lesion of the opposite optic thalamus, and that paralysis of the lower extremity is caused by an affection of the corpus striatum; but the uncertainty of these signs has been repeatedly demonstrated, and the facts appealed to in their support are, as Mr. Guthrie says, "merely coincidences when they occur." Attempts have also been made to connect certain disturbances of the intellectual faculties with injury of certain specific parts of the brain. Into this question we need not enter; abundant information on the subject exists in the former volumes of this journal: suffice it to say that experience has fully demonstrated the fallaciousness of these speculations. Mr. Guthrie, however, very properly attaches much importance to Dr. Marshall Hall's views respecting the nervous system, and conceives that they may enable us, to a certain extent, to conclude "as far as is possible what great division of the brain is most seriously injured, and more particularly with respect to the prognosis than the treatment." Thus, for example, "the loss of mobility of the iris implies an affection of the tubercula quadrigemina. Convulsions, vomiting, a drawing up of the limb not affected by paralysis, stertor, a difficulty in swallowing, strabismus, and relaxed sphincters, show derangement of the spinal functions; which is well marked when tickling the eyelashes causes no closing of the lid, the verge of the anus no contraction of the sphincter, the sole of the foot no motion of the toes." (p. 6.)

CONCUSSION. After these preliminary considerations Mr. Guthrie proceeds to treat of concussion of the brain; the mechanism of this injury, and the character of the anatomical lesions it produces, if any, have given rise to much discussion; the former of these questions he passes by in silence, the latter he considers at some length. "By the term concussion of the brain," we are told, "a certain undefinable something, or cause of evil which cannot be demonstrated, is understood to have taken place." (p. 7.) In one sense this may be perhaps true, in another it certainly is not; we can demonstrate what the *cause* of the evil is: whether any structural alteration, any anatomical lesion of the brain, has ever been recognized as an effect of that cause is another question.

With regard to the first point a reference to the mere elements of physics *demonstrates* what it is that *must* occur in concussion of the brain. The brain is a pulpy organ which exactly fills a nearly spherical bony cavity, whose parietes are elastic in a considerable though very variable degree; and if these parietes sustain any sudden change of shape, their contents must sustain a corresponding amount of compression. As any alteration in the shape of a spherical cavity must lessen its capacity, whenever any external force impinges on the cranium with sufficient violence, it must be flattened at the point of impact and expanded in some opposite direction; but these changes are, in virtue of the very cause whence they originate, of but momentary duration; the point primarily flattened by the compressing force immediately resumes its original shape, which is necessarily followed by a corresponding return of the expanded portion of the cranium to its previous dimensions. These oscillations may occur several times in rapid succession, their number and extent depending on the elasticity of the cranium, and on the amount and direction of the force applied. In concussion then the entire brain sustains a series of vibrations and momentary compressions, varying in number and amount in every imaginable degree in different cases. If this explanation, which we imagined was now universally admitted, required any extraneous proof, we might appeal to the fact that when fracture of the cranium takes place, the force of the shock is frequently expended on the bone, and concussion occurs either not at all or in but a slight degree. We cannot but consider this explanation as better calculated to give us a rational view of the nature of concussion than is the following illustration offered by Mr. Guthrie: "The term concussion is very aptly and forcibly illustrated by the homely but striking expression in use in our sister country, when a man has been suddenly killed by a fall on the head, 'That the life has been shook out of him.'" (p. 7.)

Mr. Guthrie does not allude to the experiments of M. Gama respecting this point, though he refers to his work on other occasions. M. Gama, with a view to determine the direction of the vibrations caused in the brain by concussion, arranged several pieces of thread in a glass matrass filled with a solution of isinglass of nearly the consistence of the brain.

Moderate percussion on the circumference of the globe always produced a visible effect at the point struck, and vibrations were propagated to a short distance; a stronger blow caused a momentary separation of the gelatinous mass from the glass, a similar effect being at the same time produced at the diametrically opposite point, and it then resumed its original position. This double impulsion of necessity propagated the shock to the centre of the gelatine in two opposite directions, from whence, according to the laws of impulsion, it was returned to the circumference; the threads which had yielded to these two impulses, that is to say, which were carried inwards from each side, subsequently vibrated in a contrary sense, and then underwent some irregular motions. If the matrass be held with the neck down, and the extremity of the neck be struck, the threads vibrate from within outwards (the reverse of what occurs in the former case), and the gelatine is not detached at the point of the globe opposite the insertion of the neck. The vibrations, however forcible the blow may be, are always expanded from the centre towards the circumference; as a secondary effect, indeed, the vibrating motion is returned towards the centre, but in so faint a degree as to be barely perceptible.

From these experiments M. Gama concludes that if the cranium be struck at any point, which, as well as the point diametrically opposite to it, occupies the sphericity of the cranium, the mass of the brain contracts on itself, either from the point of percussion alone, or from it and the opposite side simultaneously: the secondary and contrary vibrations are subordinate to this primary effect. But if the vertex be struck the effect is different, the brain retreats from the cranium at the percussed point alone, and the base of the cranium sustains a disseminated *contre-coup*, as it were, because of the extended surface it presents. In indirect concussion, again, communicated to the base of the cranium by the spinal column, the motion is distributed from within outwards, and the brain is not separated from the cranium at any point. (Gama, Traité des Plaies de Tête, p. 101.) It must be admitted that the glass matrass and its contents, and the cranium with the brain, are not circumstanced identically, but it strictly flows from the laws of physics that the effects of percussion on each must be the same within some degree of approximation which we cannot pretend to determine.

But do these contractions and vibrations of the brain leave any visible sign of their occurrence? In the vast majority of cases certainly not. Whatever change they may effect in the structure of the brain, that change is most commonly, if not always, inappreciable by our senses. The substance of the brain may of course be lacerated, a circumstance which is easily understood; but it has also been supposed that when concussion occurs in an extreme degree the mass of the brain may be diminished in volume so as to imperfectly fill the cavity of the cranium. Mr. Guthrie cites the cases and authorities on which this opinion rests. The first case is the well-known one recorded by Littre. The second is mentioned by Sabatier. The case of Hœnel, alluded to by Morgagni, is generally considered as another example of the occurrence; but Mr. Guthrie, by referring to the original authority, (Commerc. Literar. Nor. An, 1741. Hebd. 14,) shows that it does not bear on the point. Richerand and Delpech are said by some to have recorded cases of the kind, but, as Mr. Guthrie observes, they only refer generally to the observations of their predecessors. O'Halloran, of Limerick, says, "I have *sometimes thought* the brain did not completely fill the cavity of the skull." Dupuytren, according to Mr. Guthrie (p. 9), merely "thinks that the brain may have less consistence, more or less disposition to diminish in size, and may be readily torn by the slightest effort." But Dupuytren's testimony is much more positive than would be supposed from the passage we have just quoted from Mr. Guthrie's work. After observing that the brain presents in cases of concussion no trace of contusion, disorganization, &c. he says, "This organ has merely lost its consistence, and the slightest effort suffices to tear it. If after death from apoplexy we open the cranium, not with the hammer, but with the saw, we shall find that the volume of the brain seems to exceed that of the cavity which contains it, or, at least, it retains its natural volume and shape. After concussion, on the contrary, the brain collapses, contracts on itself, (*s'affaisse, revient sur lui-même,*) and tends to occupy less space. The reason of this is, that in concussion it contains less blood, and deprived of action and stimulus it shrinks (*tombe en affaissement.*)" (Leçons Orales, t. ii. pp. 496-7.) From this passage Dupuytren must, we think, be ranked amongst those who bear the most positive testimony to the occurrence of the anatomical lesion in question : Mr. Guthrie, however, regards its occurrence with extreme doubt, and more than doubts its connexion, supposing it to occur, with the phenomena of concussion.

"Chopart," he observes, "believes that Littre's case was much exaggerated; in which opinion I fully coincide, and place little or no reliance on the statement made by him, and so implicitly admitted since his time. I am not willing to believe that a diminution of the size of the brain, or its subsidence from the inside of the bones of the cranium, is more than an accidental circumstance which may or may not be dependent on the shock which takes place, although it be followed by immediate death. In thus expressing my dissent from a received opinion, I do so solely with the hope of stimulating future investigators to closer inquiry on this point of so much interest." (p. 9.)

It is not easy to determine what Mr. Guthrie's opinion on this subject is. On the one hand he seems to admit that a diminution of the size of the brain may occasionally occur in concussion as an accidental circumstance; while on the other he seems to place "little or no reliance" on the statements of those who aver that they have seen such diminution. Mr. Guthrie may have excellent reasons for rejecting the testimony of Littre, Sabatier, and Dupuytren, but as he has not specified those reasons we cannot pretend to estimate their value. How it is that concussion of the brain can cause a diminution of the size of the organ, is, we admit, very difficult to be explained, but perhaps we can conceive its occurrence in the following way. We have seen that, during concussion, the mass of the brain contracts on itself, the quantity of fluids circulating in it must therefore be lessened at the moment, and if death ensues on the instant, its bulk may remain permanently diminished.

Mr. Guthrie observes that "It is impossible to calculate upon what the extent and nature of the shock may be, which gives rise to a fatal concussion or rupture of the brain," (pp. 9-10,) and instances two cases in which individuals fell from great heights and sustained no such injury. The considerations we have just entered into enables us, we conceive, to explain such apparent anomalies. The occurrence of concussion does not depend merely on the shock an individual may be subjected to, but also on the force being applied in such a direction that it shall be propagated directly to the brain, and the more nearly it is propagated in a direction perpendicular to any point on its surface, the greater, cæteris paribus, must be its effect. Mr. Guthrie alludes to the notion "that if the fall be from a sufficient height, the brain will recede from the cranium, and a vacuum be formed which may be of longer or shorter duration," &c., and of course rejects it as a groundless speculation.

The symptoms and treatment of concussion are dwelt on at considerable length, and treated in a manner which leaves little to be wished for, save the minor virtues of a more methodical arrangement, and a clearer and more compact style. It would be perfectly useless to give a formal abstract of this portion of the work, as, however good in itself, it contains of course little that is not familiarly known. We shall, therefore, confine ourselves to such observations as may enable the reader to form an estimate of the manner in which Mr. Guthrie treats his subject.

Concussion of the brain may exist in very different degrees, and the symptoms vary exceedingly, not only in kind, but in intensity; it is, therefore, very convenient as regards the study both of its symptoms and treatment to consider it in its several degrees of intensity. Such distinctions must of necessity be somewhat arbitrary and vague, and Dupuytren has perhaps given us the best example of such imperfect classification of the affection. Mr. Guthrie does not adopt any such division, but describes a well-marked case in which all the symptoms are supposed to exist to a rather aggravated amount, and traces it through all its stages of collapse and reaction, ending either in rapid recovery, or passing into congestion and inflammation.

During the first stage of concussion Mr. Guthrie warns us against introducing "strong drinks" into the mouth, as, if the patient were so far recovered as to make any attempts to swallow "they might possibly enter the larynx and destroy him." (p. 12.) According to Dupuytren and Mr. Tyrrell, "in all cases of coma the act of swallowing can be excited, provided the solid or liquid be placed in contact with the fauces;" but Mr. Guthrie observes that "the first insensibility of concussion, and that which follows coma are different states." Dupuytren, however, does not limit the statement to cases of coma, but expressly makes it with regard to concussion. (Leçons Orales, p. 493.) We do not, however, deny the value of Mr. Guthrie's caution, which we think worthy of being borne in mind, though in the many severe cases of the affection we have witnessed we have never seen any inconvenience of the kind alluded to. We are also warned against causing the patient to incautiously inspire very irritant gases, such as strong ammonia, as being liable to cause subsequent inflammation of the nares and throat, and it might have been added of the larynx and trachea; any serious mischief is not perhaps very likely to result, but in several instances undoubtedly the free inhalation of ammonia or chlorine during insensibility from various causes, has produced dangerous and even fatal inflammation of those organs.

Vomiting is a symptom to which much importance has been attached in concussion. Mr. Guthrie thinks it is most apt to supervene when the symptoms begin to amend, and that "it is one of the earliest and most satisfactory symptoms of returning sensibility." It is curious to contrast the statements of the most eminent practitioners on such points: Sir A. Cooper, (Lect. by Tyrrell, vol. i. p. 254,) enumerates vomiting amongst the very earliest symptoms of concussion; but subsequently makes a statement partially coinciding with Mr. Guthrie's: "A fit of vomiting, by forcing the blood through the brain, will sometimes almost immediately restore the functions of the mind and body." (p. 262.) Sir B. Brodie says, "sickness and vomiting for the most part are early symptoms, and seldom continue after the patient has recovered from the first shock of the accident." (Med. Chir. Trans. vol. xiv. p. 239.)

"The pupils," according to Mr. Guthrie, "are for the most part in a medium or contracted state." (p. 13.) In a note he mentions and dissents from Dupuytren's opinion, that the pupils are dilated in concussion and contracted in compression. Sir A. Cooper rather coincides with Dupuytren: he says "the pupils of the eyes are generally natural; but if changed, both are a little dilated, or sometimes only one." (Op. cit. p. 254.) Sir B. Brodie's experience leans the other way: "the pupils contract on exposure to light, and are sometimes more contracted than under other circumstances." (Loc. cit. p. 338.) For our own part we have found Sir B. Brodie's statement most generally consonant with our own observation, but have not unfrequently seen the pupils dilated in concussion.

Augmented pulsation of the carotids has been mentioned by Sir A. Cooper as one of the occasional symptoms of concussion; Mr. Guthrie gives a case (p. 17,) to show that it is not a more certain sign of concussion than of compression. We are not aware that any one has considered this as a diagnostic sign between the two affections, and at all events Mr. Guthrie's case *proves* nothing, as there was no post mortem examination, though we admit that the case was almost certainly one of compression of the brain.

Mr. Guthrie examines the question whether any positive distinctive signs exist between compression and concussion, and arrives, we need hardly say, at a negative conclusion. His statements on this head do not contain anything new, but we transcribe the following passage both as a fair specimen of Mr. Guthrie's manner, and because some valuable practical points are well put. It is not perhaps superfluous to observe that Mr. Guthrie seems to consider some of the observations we are about to quote as original and peculiar to himself, but this is probably a mere inadvertence:

"The deviations which take place from the usual and ordinary modes of breathing, are supposed to offer distinctive signs of the nature of the injury which has taken place. They are, I fear, equally uncertain signs; they mark the degree of injury, and perhaps the part injured, rather than anything else. Stertorous breathing has always been considered a sign of extravasation causing compression of the brain. I have, however, seen many cases of slight extravasation with partial loss of power of one half of the body, accompanied by great numbness, without any stertor in breathing; although I have never seen a well marked case of large extravasation without it, or another peculiarity of breathing which is less thought of, although an equally characteristic and dangerous sign of such mischief having taken place when it is permanent; I allude to a peculiar whiff or puff from the corner of the mouth, as if the patient were smoking; and which, when observed among other urgent symptoms, is usually followed by death. Stertorous breathing, and the whiff or puff at the corner of the mouth are presumed to indicate an injury to the cerebro-spinal axis as well as to the cerebrum; but whether the injury is direct or indirect is uncertain, although it is frequently accompanied by extravasation or laceration. When the breathing is oppressed or laboured or heavy, neither extravasation nor lesion to any extent can in general be discovered after death. . . . Nevertheless there can be no doubt that a temporary stertor or puff at the corner of the mouth may exist, as a consequence of too great an abstraction of blood." (pp. 17, 18.)

We might point out several omissions in the history of concussion. Thus coldness of the surface is not enumerated among the symptoms of its early stage, and there is no allusion to the temporary or even permanent partial impairment of the memory or other faculty which sometimes follows it. Pain in the head is not once mentioned, even in the account of the third or inflammatory stage of the affection, and we could have wished that the very insidious way in which inflammation of the brain frequently supervenes had been noticed; the more advanced symptoms of the affection are enumerated, and that imperfectly, and it is added "inflammation of the brain is now *fully* established, and must be subdued." (p. 16.) This we fear will in most cases be much more easily said than done; if the cerebral inflammation be not detected and subdued before it is "*fully* established," the chances of death much outweigh those of recovery.

The observations on the treatment of concussion are judicious, but given in a very disconnected way. We think that sufficient importance is scarcely attached to the use of mercury when concussion is passing into its third or inflammatory state. That Mr. Guthrie is fully aware of its great utility, sufficiently appears from the following passage, but then these few lines contain literally all that is said respecting this important point of practice: "In all these and other more desperate cases, the effect of mercury, provided it had been early and rapidly administered, may yet be decisive. Calomel, combined with another and not less important remedy, opium, ought to be given every two or three hours until the effects of both are fairly induced." (p. 25.) We must also take exception to the statement that opium is in these cases not less important than calomel, and that the patient should be brought fully under its influence; we should certainly regard with apprehension so very liberal an use of opium in those cases, and merely employ it, if at all, with a view to accelerate the action of the mercury on the system.

In the twenty-ninth and following pages, Mr. Guthrie adverts to an important class of cases, which are however of unfrequent occurrence, those in which "mania supervenes on the injury." Mr. Guthrie suspects that this occurs only when the sufferer has an hereditary predisposition to insanity, and rarely unless he has shown some previous symptoms of such derangement, and does not advert to Sir B. Brodie's opinion, "that furious delirium and convulsions occur after an injury of the head under nearly parallel circumstances: the former symptom, like the latter, may be produced by pressure on the brain, not however by such a degree of pressure as threatens completely to annihilate the functions of that organ, but by that smaller degree of pressure which operates merely as a source of irritation." (Op. cit. p. 375.) Mr. Guthrie recommends the use of opium in those cases: "The value of the treatment," he states "is entirely dependent on the substitution of opium and morphia, with a moderately nourishing diet, for bloodletting; I have not met with an instance in which it was necessary to give wine or other stimulants, but I can readily conceive that cases requiring them may occur in very irritable persons, or who may have been in the habit of indulging in their use." (p. 32.) Sir B. Brodie, on the contrary, tells us that "when a patient is affected with furious and roving delirium, blood should immediately be taken from the arm, and if possible in a full stream. I scarcely remember a single case, in which delirium of this kind, occurring soon after a blow on the head, did not yield to a copious bloodletting." (Loc. cit. p. 422.) Perhaps Mr. Guthrie may mean to speak of a different class of cases from those referred to by Sir B. Brodie; but as he simply uses the term "mania," we cannot determine this. Three cases indeed are given to illustrate the value of the treatment recommended; but in one no symptom of mania or delirium were present, and in another the patient is described as being "violent," and requiring "the restraint of a tight jacket." Greater precision in defining the therapeutic indications relative to so important a point of practice would have been highly desirable.

COMPRESSION. The account of compression of the brain is prefaced by some considerations relative to the opinion especially advocated by Munro (Secundus), and Sir C. Bell, that the proper substance of the brain is incompressible. It must, we take it, be admitted that the molecules of the brain are compressible in but a very slight degree, probably not more so than water is. Putting the theoretical question aside, however, the following considerations must ever influence the practitioner:

"Considering that we can often see the natural and ordinary size of the brain diminished under pressure, and that certain symptoms, such as insensibility, syncope, convulsions, paralysis, are consequent on this state, and are relieved by the removal of the pressure and the restoration of the compressed brain to its ordinary state, we may safely conclude that some derangement takes place in its integral parts, which may be best understood by the word compression. . . . When we see a patient lying in a state of insensibility with a fracture of the cranium, immediately recover his senses, after the application of the trephine and the removal of a large coagulum of blood, we are apt to conclude that the coagulum of blood and the insensibility stand in the relation to each other of cause and effect." (p. 40.)

With reference to Sir C. Bell's opinion that compression acts not on the substance of the brain, but only on its blood-vessels, lessening their diameter, and consequently diminishing the quantity of blood circulating in the organ where its functions are interfered with, Mr. Guthrie says, "it is not unreasonable to conclude that the pressure had occasioned the insensibility, and that this did not depend alone upon some few vessels containing less blood than usual." For our own part we quite agree with Sir B. Brodie, "that, although we admit the substance of the brain to be incapable of being compressed into smaller compass, yet that the effect of all pressure on it must be, and is, to alter the position and relative situation of the delicate fibres of which its minute structure is composed, and that we need seek no further explanation of the symptoms which are met with in these cases." (Loc. cit. p. 334.)

Mr. Guthrie notices the experiments of Serres and Flourens on the influence exerted on the functions of the brain by blood extravasated within the cranium. Serres trephined a dog over the longitudinal sinus, and transfixed the sinus with a narrow bistoury; the animal retained its natural liveliness, though on opening the cranium some hours after the experiment, large coagula of blood were found on the surface of the brain. This experiment was frequently repeated and uniformly with the same result; no stupor, none of the symptoms of compression were produced. M. Flourens objected to these experiments that blood could only compress the brain while it was itself compressed by the cranium; and, by operating on young animals, was enabled to perforate the sinuses of the brain without trephining the skull; and the hemorrhage thus produced was followed by symptoms of compression, which were immediately relieved on removing a portion of the cranium,—an observation which is illustrated by a remark of Mr. Keate, mentioned page 56, but not alluded to by Mr. Guthrie in connexion with these experiments: "Mr. Keate has invariably remarked that the symptoms dependent on extravasation have been less severe in the first instance, in proportion as the separation of the edges of the fracture has been greater one from the other, or when the sutures have yielded to the shock, and been separated." The degree of pressure unquestionably exerts a material influence on the symptoms. Sir A. Cooper states in his Lectures, that having trephined a dog and pressed on the brain with his finger, the animal at first suffered no inconvenience, but on the pressure being gradually augmented, became affected with complete coma; M. Malgaigne's experiments, (which Mr. Guthrie does not notice,) performed by injecting water into the cranium of rabbits, go to establish the same point, viz., that mere compression unaccompanied with lesion of the brain produces no bad effects, unless it is exerted to a very considerable degree. (Anat. Chir. v. i., p. 315.) But however conclusive such experiments may appear, they do not enable us to clear up the difficulties that occur in practice, as Sir B. Brodie has observed: "It is difficult to explain wherefore the symptoms to which it (compression) gives rise are sometimes slight, and at other times urgent, although occurring under circumstances apparently similar. A depression of bone, which in one instance produces comparatively little effect, in another case occasions a manifest destruction of sensibility, and the same observation may be made respecting extravasated blood. Every practical surgeon must have observed that there are differences in the symptoms produced, which are not to be accounted for by any differences in the quantity of pressure, nor in the particular part of the brain which is affected by it." (Loc. cit. p. 344.) Serres, Gama, and other French surgeons seek to escape from the difficulty by saying that the symptoms commonly attributed to compression, are rather produced by irritation of the brain; but this explanation does not seem very satisfactory.

The account of the symptoms of compression of the brain is given at great length, and we may add with great judgment and truth, evidencing extensive, and what is much better, accurate observation. We shall make no apology for presenting the following rather lengthy extracts to our readers, which we select as specimens of Mr. Guthrie's minuteness and fidelity of detail. The observations on convulsions also contain some interesting and valuable practical remarks, which further induces us to extract them:

"Paralysis of one side of the face and hemiplegia are common; paraplegia is more rare. In both kinds of palsy, one part in one limb may be more completely affected than another, in which convulsive twitchings are sometimes present, as well as a frequent drawing up of the limb of the unaffected side.

Tickling the soles of the feet or the palms of the hands will sometimes cause retraction of the toes or fingers, when the limbs are apparently motionless; pricking them gently with a pin will often give rise to convulsive startings and tremblings of all the muscles of the extremity when tickling fails, showing that the capability to move the part remains, although the will to do so is wanting. The leg or arm is sometimes drawn towards the body when separated from it; it more often falls from the hand as if it belonged to the body of a dead person; the muscles are occasionally more stiff and rigid, and some power of motion remains, although little of sensation; sometimes sensation is perfect when motion is lost, and sensation may be lost on one side, and motion on the other." (p. 43.)

"Convulsive actions of the muscles or positive convulsive fits, are always important symptoms; yet they seem in some persons to be dependent on idiosyncrasy, particularly when they appear early and after the loss of blood, in which cases they are less dangerous. They occur at different periods after the receipt of the injury, and have been supposed to depend in general upon laceration of the substance of the brain, although experiments on animals would seem to show that they may be caused by irritation of the cerebro-spinal axis within the skull, when the patients are more likely to recover. They have been observed to accompany these injuries from the earliest antiquity, and to appear particularly on the side opposite to that which is paralytic, so as to give rise to the idea that paralysis was dependent on injury of one side of the cerebrum, and convulsions of the other. When the effect of the injury is so great as to prevent the transmission of sensibility along the nerves to the muscles, the paralysis is complete, and, I suspect, convulsive twitches do not then take place, although they frequently precede and may in many cases be considered as premonitory signs, whilst the evil which gives rise to the paralysis is gradually accumulating. When the paralysis is not complete, I have frequently seen that side affected by slight convulsive twitches, whilst the other suffered from well marked spasms; leading to the belief, that whilst paralysis is an affection of only one half of the brain of the opposite side, or of half of the spinal marrow of the same side, convulsions are the effect of a more general irritation, capable however of being confined to a part; for partial convulsive motions do very frequently occur without any paralysis accompanying them on the opposite side, and I have not seen these convulsive actions occur, as far as I can recollect, where both sides have been paralytic from injury of the head, although spasms and twitches are symptoms of daily occurrence in paraplegia from disease of the spine. I have met with several cases in which the convulsions have ceased, and the patients recovered after the removal of a portion of bone which was irritating the brain, but convulsions have generally been the forerunners of death when the seat of injury was unknown, and this assistance could not be given. . . . Convulsions, it must be remarked, are among the most common symptoms of inflammation of the membranes of the brain, although they are frequently wanting. They may be expected to take place about and after the fifth day in injuries of the head, when inflammation of the brain is about to extend to or become continuous with the neighbouring parts, and may be more or less severe, varying from a state of partial trembling of a limb, to that of general agitation and restlessness of the body generally; from a slight irregular movement of the eyelids or muscles of the face, to the more marked spasmodic startings of the whole of one side, grinding of the teeth, and contraction of the limbs. Sir B. Brodie has well shown that they may exist at a late period independently of inflammation, 'being aggravated by any additional abstraction of blood, and subsiding on the patient being allowed to take some more substantial nourishment than that which had been allowed him previously.' They would seem in these cases to be dependent on the same or similar causes to those which gave rise to them after the loss of too great a quantity of blood in the first instance, and to be relieved or removed in a similar manner. It is far different with those convulsive movements which, at a late period, become nearly permanent or rigid spasms, resembling tetanus, in which the body is drawn in different directions forwards, backwards, or to one side. They are for the most part the forerunners of death; fortunately they are seldom present except in very hot weather, and are not even then of very frequent occurrence. Examination after death in such cases has shown nothing discoverable beyond inflammation of the pia mater, and an effusion of fluid, generally purulent, on the surface of the brain or in its ventricles, or between the pia mater and tunica arachnoides." (p. 50.)

When a patient retains his consciousness and faculties unimpaired after receiving injury of the head, but after the lapse of a short period becomes heavy, drowsy, stupid, unwilling to move, and gradually gets insensible, it is usually considered that extravasation of blood has taken place; a short interval of consciousness between the receipt of the injury and the supervention of the symptoms of compression, as a certain, though doubtless rather rare, distinctive sign between concussion and compression, and conclusive as to the occurrence of the latter affection. Mr. Guthrie twice states (pp. 23, 56,) that under such circumstances, "extravasation has not always taken place," and the symptoms of compression may arise from mere congestion.

"If," he says, "the loss of a moderate quantity of blood should relieve such a person, it shows that congestion had occurred, perhaps on the surface of the brain, under the injured spot; recovering from which by the unassisted efforts of nature, he would still be liable to inflammation. I have repeatedly seen a small bleeding from an incision made to allow a complete examination of the part in such a case, cause the restoration of the patient to his natural state. A return of untoward symptoms during the progress of the case does not always indicate essential mischief, and will be removed, if of a temporary nature, by a further moderate bleeding, by purgatives, &c.......If the loss of blood should not relieve the symptoms, the case is probably complicated by an extravasation having taken place between the dura mater and the bone, and even in, or on the surface of the brain." (pp. 56-8.)

Four cases are given in support of this opinion, (pp. 23-4, 57,) in which the patients recovered under the circumstances in question, after bloodletting. A case is also quoted from Le Dran (16th Observation, p. 57, note,) exactly parallel to those mentioned by Mr. Guthrie, save that the patient ultimately died; and on dissection there "were found several small spots of coagulated blood in different parts of the brain, one of which was as large as a nut." We think that this case, quoted by Mr. Guthrie himself, should have induced him to modify his opinions on this point. We cannot pretend to say that the supervention of symptoms of compression following an injury of the head after so short an interval of consciousness that inflammation has not had time to set in, may not occasionally depend on congestion, but, we ask, where is the proof? The test mentioned by Mr. Guthrie, the recovery of consciousness after the loss of blood, is evidently fallacious. In Le Dran's case this did occur, and yet dissection proved that extravasation of blood had taken place. Moreover, the insensibility produced by actual compression on the brain, does not by any means always remain constant in amount. To again avail ourselves of the words of Sir B. Brodie, (loc. cit. p. 346,) "at one time it may be perfect, then the patient may show some signs of consciousness, and then relapse into a state of stupor. It may be observed that there is especially an increase of insensibility after bloodletting." The cases cited then by Mr. Guthrie may have been, and, looking to Le Dran's case, probably were, cases of extravasation of blood to a small extent, in which bloodletting relieved the symptoms to a greater extent than it usually does, and at the same time arrested the hemorrhage, so that the amendment was permanent.

With reference to fracture, accompanied with rupture of the middle meningeal artery or its branches, five cases are given (pp. 59-61), in each of which there was an interval of sense between the injury and the occurrence of symptoms of compression ; four of the patients recovered; in each of them the brain was depressed, but recovered its natural level when the compressing cause (the coagulum of blood) was removed; in the fifth case the brain did not regain its level, and the man died. The same circumstance, followed by a like fatal result, occurred, we may observe, in Mr. Abernethy's 12th case. (Surgical Works, vol. ii. p. 36.) Mr. Guthrie, we presume, has observed several cases of the same kind, as he says (p. 59), "I never saw the symptoms mitigated, or the persons in any way relieved, when the brain remained depressed after the blood had been removed;" and we suppose so general a statement would hardly be made from the experience of a single case. It is perhaps superfluous to say, that Mr. Guthrie recommends, whenever symptoms of compression follow a wound over the track of the middle meningeal artery, that "the bone should be immediately examined; and if there should be no obvious fracture, and relief cannot be obtained by the abstraction of blood, the trephine must be resorted to as a last resource; for if there be truth in the statements so confidently made of fracture of the inner table of the bone from concussion of the outer without fracture, it is here especially that we may be permitted to look for it." (p. 58.) And again: "The rule in surgery, to remove the bone in such cases, appears to me to be absolute." (p. 61.) It would appear, from the first of these passages, that Mr. Guthrie is somewhat doubtful about the possibility of the middle meningeal artery being ruptured, without the skull being either fractured or depressed. He does not, on this point, allude to the two remarkable cases quoted by Mr. Abernethy from Mr. Hill and Mr. Latta, in which this actually occurred, though he subsequently mentions Mr. Hill's case, as being "the most remarkable for its severity, and for the recovery of the patient," with which he is acquainted.

The possibility of the occurrence of fracture of the cranium by *contre-coup*, has given rise to much discussion. Mr. Guthrie gives a review of the best-attested cases in which it has been observed, or supposed to have been observed. What his own opinion may be on the subject does not appear, but he observes, "whatever may have happened formerly, there is so little proof of such an occurrence having taken place in later years, that the accident is in general altogether overlooked by writers on injuries of the head." (p. 65.) This observation, of course, does not refer to a fracture of the base of the cranium from a blow on the vertex, or on the back part of the head.

With respect to this latter accident, Mr. Guthrie dissents from the opinion of Mr. Earle and Sir C. Bell,—that the fracture of the base of the cranium depends on the occiput being forcibly impelled against the atlas, and thinks that it arises from "the superincumbent weight of the body pressing on the unsupported, flat, and thin base of the skull," (p. 65,) and seems to doubt that it can occur from a blow on the vertex. We do not see that Mr. Guthrie's explanation makes the matter very clear: the question is,—How is the force (whether it be the superincumbent weight of the body, or anything else,) propagated so directly to the base of the cranium as to produce fracture in that situation? And this question has, we conceive, been answered by a reference to the mechanism of the cranium. If a force be applied to the vertex, the superior borders of the parietal bones resist displacement downwards, inasmuch as their inferior borders cannot be thrown outwards, in consequence of their being supported laterally by the overlapping of the squamous portions of the temporal bones; while the temporal bones, as M. Malgaigne has pointed out, are themselves supported by the zygoma, which constitutes on each side a true buttress, sustained by the superior maxillary bone. A check then applied to the vertex, is directly transmitted to the temporal bone, and propagated through its petrous portion to the posterior part of the body of the spheroid bone, the very parts which most fractures of the base of the cranium traverse. The precise direction of the fracture must, of course, vary with the direction in which the force is transmitted, but what has been said sufficiently explains the general theory of its occurrence.

Though fractures of the base of the cranium are almost uniformly fatal, because they are usually complicated with other more serious injuries, it is yet generally admitted that recovery from the accident occasionally occurs. Four cases are given (p. 66, et seq.) as examples of recovery from this accident; two of them probably were so, but in the absence of a dissection cannot be unreservedly admitted as such: in the other two cases, there was an opportunity for a post-mortem examination at a subsequent remote period, and the previous occurrence of fracture of the base of the cranium was placed beyond doubt. It is supposed that bony union does not occur in fractures of the base, as it does in simple fractures of the vault of the cranium, but that they terminate, in the event of the patient surviving, in a false articulation as it were: no allusion is made to this point in the account of the dissection, though some incidental expressions lead us to suspect that bony union had not taken place in either case.

Mr. Guthrie incidentally notices in the report of a case (p. 70,) that very curious symptom, "the discharge of a fluid resembling water" from the ear, a symptom "which," he observes, "is usually very dangerous, as it flows from the sac of the arachnoid membrane." We quite agree that this is a symptom of very bad omen, as we have witnessed but one recovery amongst a considerable number of cases in which it occurred; but we cannot coincide with our author as to the origin of the fluid, inasmuch as we possess positive evidence from dissection, that it occurs when the membranes of the brain are intact, and the cavity of the arachnoid consequently not opened. The researches of Laugier also, if we recollect right, for we have not his paper at hand, have already established the same fact.

Mr. Guthrie has never met with a case in which the inner table of the cranium was fractured, the outer table at the same time remaining uninjured, and his observations respecting this very rare occurrence require no particular remark. He has, however, collected most of the recorded cases of the accident, which will be found useful and convenient for reference. The same remark nearly applies to the observations on those remote ill effects that occasionally follow wounds of the head, with or without fracture or depression of the bone, and sometimes call for operative interference; a number of very interesting cases recorded by others are collected, and one is added from Mr. Guthrie's own practice, but no general conclusions are deduced, and no rules are laid down for the guidance of the practitioner, though the cases collected by our author perhaps afforded sufficient materials for at least an attempt at so doing. The case operated on by Mr. Guthrie himself, though there are several analogous to it amongst those he has cited, deserves notice. A healthy girl received a blow on the left side of the head from a stone, which was followed by acute pain, which permanently marked the seat of injury, and after a week she began to lose the power of moving the right arm. Matters continued thus during twelve months, when the paralysis of the arm suddenly increased. No further change occurred during the eleven succeeding months, when she became affected with hemiplegia of the right side, impaired sight, hearing, and memory, and occasionally slow, almost stertorous respiration. All the ordinary remedies having failed to relieve the symptoms, Mr. Guthrie, two years after the injury, removed with a trephine "a disc of bone from the exact point in the parietal region to which she referred the pain. The portion of bone presented no evidence of disease......the dura mater was quite healthy, and without any very evident motion. On visiting her *an hour* after the operation, she raised the previously paralytic arm several inches from her bed, and was able to bend and extend the fingers. The pain in the head was considerably less, and her countenance, before dull and heavy, was now remarkably animated; sensation had returned in the arm, and partially in the leg." She was subsequently threatened with inflammation of the brain, which was averted by bloodletting, and "in the course of three days the paralysis had completely disappeared, sight and hearing again became perfect, and, after passing through a speedy convalescence, she quitted the hospital completely recovered." Some relapses of pain and uneasiness in the head subsequently occurred, but she considered herself "to have been cured by the operation; although," observes Mr. Guthrie, "I find it difficult to say in what manner it was effected, or why the removal of the bone, which was in a perfectly natural state, should have given relief." (p. 85.)

"The inner table is sometimes," Mr. Guthrie observes, "broken in a peculiar manner, and to which I believe attention has only been drawn by myself in my lectures, since trepanning has ceased to be the rule of practice in all cases of fractures. It occurs from the blow of a sword, hatchet, or other clean cutting instrument, which strikes the head perpendicularly, and makes one clean cut through the scalp and skull into the brain. This kind of cut is usually considered as a mere solution of continuity, and not as a fracture; the bone being apparently only divided, with scarcely any crack or fissure extending beyond the part actually penetrated by the instrument." (p. 85.)

When the wound only implicates the outer table or diploë, it should be treated as a simple incised wound of the scalp, but "when the sword or axe has penetrated as far as, or through the inner table, the case is of a much more serious nature; for this part will be broken almost always to a greater extent than the outer table, and will be separated from it, and driven into the membranes, if not into the substance of the brain itself." (p. 86.) Mr. Guthrie accordingly recommends that

"These cases should all be examined carefully. The length of the wound on the top or side, or any part of the head which is curved and not flat, will readily show to what depth the sword or axe has penetrated. A blunt or flat-ended probe should in such cases be carefully passed into the wound, and being gently pressed against one of the cut edges of the bone, its thickness may be measured, and the presence or absence of the inner table may thus be ascertained. If it should be separated from the diploe, the continued but careful insertion of the probe will detect it deeper in the wound; a further careful investigation will show the extent in length of this separation, although not in width, and will in all probability satisfy the surgeon that those portions of bone which have been thus broken and driven in, are sticking in or irritating the brain." (p. 86.)

When the nature of the case has been ascertained by the examination thus recommended, the question arises what should be the practice, and Mr. Guthrie replies,

"There can be no hesitation in answering, that in all such cases the trephine should be applied, although no symptoms should exist, with a view of anticipating them ..... A patient very often survives a mere depression of the skull; he may, and occasionally does survive, a greater depression of the inner than of the outer table; but I do not believe that he ever does survive and remain in tolerable health after a depression with fracture of the inner table, when portions of it have been driven into the dura mater. If cases could be advanced of complete recovery after such injuries, I should not consider them as superseding the practice recommended, unless they were so numerous as to establish the fact, that wounds of the dura mater and brain by pieces of bone are not extremely dangerous. I have referred purposely to many cases in which a cure was effected after a lapse of time, by the bone being removed, but they rather support than invalidate the principle I have inculcated ..... I maintain that the practice should be prompt and decisive in every instance in which the surgeon is satisfied that there is not merely a slight depression or separation of the inner table, but that several points of bone are driven into the dura mater." (p. 94.)

In discussing the treatment of depressed fracture Mr. Guthrie first adverts to the well-known fact that these injuries are less serious in the child than in the adult, and seems to consider that this fact has assisted in misleading those who advocate *La chirurgie expectante* in those cases at other periods of life; thus he observes, " If the records of surgery for the last twenty years be carefully examined, it will be found that the greater number of successful cases of recovery from depression, or from fracture and depression of the skull, which were not trephined, were in young persons, a fact which is deserving of the most serious attention," (p. 102;) and again, " By far the greater number of cases in which recovery has taken place after fracture and depression of the skull, with injury of the brain and even loss of its substance, have occurred in children or in persons under the adult age," (p. 112;) and he concludes, " that a continuance of the most urgent symptoms can alone authorize the application of the trephine in children and young persons under fifteen or sixteen years of age," (p. 102;) and that " less frequent recourse may be had to the aid of operative surgery in order to prevent mischief than in adults, even when the bone is fractured as well as depressed," (p. 112,) a rule of practice already fully established and generally admitted.

In the case of adults, however, Mr. Guthrie is a decided advocate for early interference. Whenever a marked depression of the cranium occurs in a person of mature age, he recommends us, if it be a simple fracture, to divide the scalp, and ascertain the nature and extent of the depression, as he thinks the difference between a compound and simple fracture "has been much exaggerated; so much so, that he places no reliance on the supposition that there is more real danger in a case of fracture with depression, in which the scalp has been divided, than when it has been only bruised and not divided," (pp. 103-4,) a doctrine against which we must utterly protest. When the depressed bone has been thus exposed, we are told to proceed according to circumstances. Mr. Guthrie considers it "imperative to remove at once all portions of bone or foreign substances which may have or may be supposed to have penetrated the dura mater in adults, although no symptoms of compression should be observed." (p. 117.) Where the dura mater is not wounded, "there is more room for doubt; and the surgeon must then decide, after the best estimate he is able to make of the probable evil which will occur from allowing the broken or depressed portions of bone to remain." (p. 112.) Whenever the bone, however, presses considerably or "unequally," to use Mr. Guthrie's own word, on the dura mater without wounding it, he seems to recommend that it should be elevated; we say seems, for his expressions are occasionally somewhat vague. Such is the summary of our author's doctrines relative to this questio vexata in surgery; any commentary on our part would be superfluous, as our readers must be familiar with all that has been said by Larrey, Sir A. Cooper, Sir B. Brodie, and others, more or less favouring the rules of practice adopted by Mr. Guthrie, and equally so with the opinions of those who, with Dupuytren, Mr. Colles, and Mr. Lawrence, recommend us to postpone operative interference until the occurrence of symptoms shall imperatively demand it. We may however say thus much, that while we quite agree with Mr. Guthrie in the propriety of removing any portion of bone which wounds the dura mater, we dissent from his recommendation to operate in a case of mere depression without symptoms of compression.

The observations on flap-wounds, ecchymosis, and erysipelas of the scalp, require no particular examination; though brief, they are excellent so far as they go. A case is mentioned where a portion of bone was sliced off with and adherent to a flap of the scalp; and on the parts being replaced in situ, both flap and bone adhered. (p. 96.) Some similar cases recorded by others are mentioned, but no allusion is made to the four remarkable cases mentioned by Larrey, in which sabre wounds sliced off, with the flap of the scalp, not merely a portion of the entire thickness of the cranium, but also of the meninges and of the brain, and the parts being replaced union and recovery ensue. (Clinique Chir., t. i. p. 140.)

The formation of pus between the dura mater and cranium seems to have been a much more common consequence of injuries of the head in the time of Pott and Deane than at present; this Mr. Guthrie very properly attributes to the rigid antiphlogistic treatment and regimen now adopted in cases of injury of the head.

Mr. Guthrie does not consider wounds of the dura mater by any means so dangerous as they were supposed to be by Hunter, Sir A. Cooper, and we believe we may add, most British surgeons; and thinks that the arachnoid membrane, when wounded, is not so liable to diffused inflammation as might be expected, "in consequence, probably, of the more equal pressure that is kept up within the skull than in the chest or abdomen." (p. 128.) He thence considers that British surgeons may, perhaps, be in error in not more frequently puncturing the dura mater, to admit of the discharge of blood or matter, and recommends this practice under the circumstances described in the following extract:

"I have seen, on the removal of a portion of the bone by the trephine, the dura mater rapidly rise up into the opening, so as to attain nearly the level of the surface of the skull, totally devoid, however, of that pulsatory motion which usually marks its healthy state; and an opening into it, under these circumstances, has allowed a quantity of purulent matter to escape, proving that the unnatural elevation of the dura mater was caused by the resiliency of the brain when the opposing pressure of the cranium was removed. I consider this tense elevation, and the absence of pulsation, to be positive signs of there being a fluid beneath, requiring an incision into the dura mater for its evacuation." (p. 125.)

Three cases are given in verification of these observations, two of them terminated fatally and one recovered.

The peculiarities attending gunshot wounds of the head, are adverted to briefly, yet perhaps sufficiently. Mr. Guthrie observes, that a musket-ball may sometimes, in penetrating the cranium, make a hole not larger than itself; but Larrey has observed that the orifice may be so small that the entrance of the ball would not be suspected, if we did not bear in mind that in young, or even middle-aged persons, in whom alone this can occur, the bones possess considerable elasticity, and the more elastic the bone the smaller the opening. (Clinique Chir. t. i. p. 213.)

Both at the commencement of the work (pp. 2, 3,) and again in the observations on gunshot wounds (p. 132), Mr. Guthrie maintains that wounds of the anterior part of the brain are more dangerous than those of its middle portion, and that those of its posterior part are attended with least evil. This statement is directly opposed to the opinion and experience of every practitioner with whose opinions we are acquainted, and is contrary to our own observation of the result of a considerable number of cases.

Four cases are mentioned of fracture of the skull through the orbit (pp. 137-8); the very deceptive nature of many of these cases, and the insidious manner in which the symptoms usually supervene, though well exemplified by the detail of two cases, might perhaps have been more specifically mentioned, for the benefit of the student, and even of some practitioners.

Mr. Guthrie recognizes two kinds of hernia cerebri, one that particularly described by Mr. Abernethy, "principally composed of coagulated blood;" the other, "formed for the most part of brain," as mentioned by Mr. Stanley and others; the former being much the more dangerous, and generally fatal, indeed uniformly so in our author's experience. Of the causes of the former he says nothing; that the latter are "the consequence of low inflammation of the brain, there can" we are told, "be no doubt." (p. 144.) Mr. Guthrie says that this kind of protrusion "rarely or never takes place when a considerable portion of the skull has been lost or removed, the brain being able to expand to such extent as the inflammatory impulse from within may render necessary. When the opening, on the contrary, is small, and the dura mater has not been injured, they have been seldom observed. It is then principally when the opening in the skull has been of greater extent than the size of one piece of bone removed by the trephine, the dura mater having yielded, either in consequence of injury or by ulceration, that this evil takes place; it is by no means, under proper treatment, a fatal, though it is always an extremely dangerous occurrence." (pp. 138-9.) Mr. Abernethy is the only surgeon we can recollect who agrees with Mr. Guthrie in considering that the loss of a considerable extent of the cranium does not favour the occurrence of hernia cerebri. Mr. Guthrie further states, "there can be no doubt of the formation of many of these protrusions being aided by the opening which has been made in the dura mater, which would have restrained their growth if it had been sound." (p. 144.) Sir B. Brodie, who has discussed this point, (Med. Chir. Trans. vol. xiv. pp. 396-415,) entertains opinions the very reverse of Mr. Guthrie. He considers that the removal of a portion of the cranium "may in itself be sufficient to make the patient liable to this formidable and dangerous disease of hernia cerebri;" and, in support of this opinion, appeals to cases in which, "though the dura mater was found not to have suffered from the injury, yet a *hernia cerebri* presented itself some days afterwards." We have ourselves seen the affection, both where the dura mater was wounded and where it was not, and certainly consider that Mr. Guthrie attaches too much importance to the lesion of this membrane, and too little to the absence of a portion of the cranium, as connected with the development of this affection. As respects the treatment nothing novel is advanced; the practice recommended is that inculcated by Sir A. Cooper, Dupuytren, and others,—pressure, graduated and augmented according as the patient can bear it. Escharotics are of course condemned, and yet in one of the cases appended (p. 141,) part of the treatment adopted consisted in touching the tumour with nitrate of silver.

The concluding pages of the work are occupied by some observations on the impairments of intellect, &c., that occasionally follow injuries of the head; and on the operation of trephining; on neither of which subjects is anything said requiring observation.

We have laid so very full an analysis of Mr. Guthrie's treatise before our readers, that it is scarcely necessary to add anything to the comments which we have already occasionally made. We must say, however, that we consider the work a valuable and seasonable addition to medical literature. We by no means agree in all Mr. Guthrie's doctrines, but his opinions are entitled to a candid examination, and while we retain our own views we shall not yet dogmatically pronounce his to be wrong. Did Mr. Guthrie's work do nothing but excite those, who have opportunities for the task, to re-examine the more doubtful and litigated questions connected with the treatment of injuries of the head, it would effect much good; but it does more; for, putting aside any future results it may produce, it must long remain a work valuable to the student for instruction and to the practitioner for reference, as there can be no dispute respecting the truth and value of the great majority of the doctrines and rules of practice it contains, and few positions are advanced which are not illustrated by appropriate and instructive cases. Should a second edition be called for, there are a few points which might be usefully treated of at greater length; but a much more essential improvement might be effected by the adoption of a more methodical arrangement, and a less diffuse and rambling style. It would almost appear as if Mr. Guthrie had strung together a number of detached notes, made at different periods, without much troubling himself as to the order in which he connected them. Be this as it may, defective arrangement characterizes the book; and this, together with diffuseness of style and occasional vagueness of expression, renders it sometimes difficult to detect the author's precise meaning, or to hunt out the passages relating to any particular point. Having said this much by way of censure, we conclude by cordially recommending Mr. Guthrie's treatise to the attention of our readers.

---

